# Overall Clinical Features of Type 2 Diabetes Mellitus With Respect to Gender

**DOI:** 10.7759/cureus.35771

**Published:** 2023-03-04

**Authors:** Javeria Ali, Syed Muhammad Safi Haider, Syed Mushhood Ali, Taimur Haider, Adnan Anwar, Atif A Hashmi

**Affiliations:** 1 Internal Medicine, Abbasi Shaheed Hospital, Karachi, PAK; 2 Internal Medicine, Hamdard College of Medicine and Dentistry, Karachi, PAK; 3 Internal Medicine, District Headquarter Hospital, Jhang, PAK; 4 Physiology, Hamdard College of Medicine and Dentistry, Karachi, PAK; 5 Pathology, Liaquat National Hospital and Medical College, Karachi, PAK

**Keywords:** comorbidities, gender, frequent urination, dry cracked skin, type 2 diabetes

## Abstract

Introduction

Since patients with type 2 diabetes are frequently misdiagnosed, provided inappropriate management, or poorly controlled, it is important to comprehend the wide range of clinical signs and symptoms associated with diabetes. Therefore, this study evaluated the overall clinical manifestations of patients with type 2 diabetes patients with respect to gender.

Methods

This was a multicenter, cross-sectional study that was conducted at various hospitals, using a non-probability sampling technique. The duration of the study was about six months, from January 1, 2022 to June 30, 2022. The study included 590 type 2 diabetes patients, ranging in age from 35 to 70 years. Age, gender, socioeconomic status, health status, co-morbidities, and diabetes symptoms were documented. A chi-square was applied to determine the association between overall symptoms associated with type 2 diabetes and gender. An independent t-test was applied to determine the significance level between means of demographic parameters.

Results

The study findings showed that out of 590 patients with diabetes, 310 (52.5%) were males and 280 (47.5%) were females. The male and female mean ages were 57.46±14.93 and 50.38±14.85 years, respectively, with a statistically significant gender difference (p<0.001). The prevalence of renal manifestation in type 2 patients with diabetes revealed a significant relationship (p<0.05) for both genders. The prevalence of ocular manifestations revealed a significant relationship with both genders (p<0.05) in terms of distortion and blurred vision. The prevalence of ocular manifestations revealed a significant relationship observed with both genders (p<0.05) in terms of shortness of breath, dyspnea severity, and severity of chest pain.

Conclusion

This study concluded that women with type 2 diabetes mellitus have a significantly higher frequency of muscular pain, urinary symptoms, neurological symptoms, and dermatological manifestations than men. In contrast, respiratory symptoms were significantly more pronounced in males than in females. The presence of comorbidities such as dyslipidemia significantly increased the probability of developing type 2 diabetes in both genders.

## Introduction

Over the past few decades, both the burden and prevalence of diabetes mellitus (DM) have increased significantly on a global scale. By 2045, more than 629 million people between 20 and 79 years will have diabetes, according to current reports [1]. Every 8 seconds, a person dies from diabetes, which is estimated to have killed four million people worldwide in 2017 [1]. Eighty percent of diabetics reside in lower- and middle-income nations. In most of these populations, the incidence and prevalence of DM, formerly thought to be a rare medical illness in Africa, is rapidly rising. Type 2 diabetes is the most prevalent among patients [2]. It was originally expected that by 2025, the majority of the world's population with diabetes would reside in developing countries due to rising life expectancy, an aging population, and increased urbanization. As a result, the long-term consequences connected with diabetes will continue to influence people's individual and communal health in these regions [3].

All ages and socioeconomic categories are susceptible to DM. It is described as hyperglycemia due to an absolute or relative insulin deficiency. There are two subtypes of DM type 1 or insulin-dependent diabetes (IDDM), and type 2 or non-insulin-dependent diabetes (NIDDM). The incidence of DM type 2 was 171 million worldwide in 2000, and it is predicted that the number will increase to 366 million by 2030 [4].

The intensity of symptoms depends on the type and duration of diabetes. Some diabetes patients, particularly those with type 2 diabetes in the early stages, are asymptomatic; others with high hyperglycemia, particularly in children with absolute insulin insufficiency, may experience excessive urination, thirst, increased hunger, weight loss, and blurred vision. Uncontrolled diabetes can develop ketoacidosis or, more rarely, a non-ketotic hyperosmolar syndrome that causes stupor, coma, and, if untreated, death [5,6].

Overall, men are more likely to have diabetes than women, but women are more likely to have type 2 diabetes [7]. According to the stage of reproductive life, the gender disparity in diabetes incidence is changed: more men develop diabetes before puberty, whereas more women develop diabetes after menopause and in later life. Considering the higher incidence of type 2 diabetes in women, it is critical to emphasize that males are more likely to develop forms of diabetes exacerbated by diabetic ketoacidosis or ketosis [8]. Women are protected from ketosis and ketoacidosis, and only a hypoestrogenic or protracted ovulatory state renders this protection ineffective [8].

It is also noteworthy that the association between changes in glucose homeostasis after meals and an increase in visceral fat, and the presence of this tissue in women only augments insulin resistance. To achieve the desired glycemic target, oral hypoglycemic medications are becoming increasingly therapeutically necessary based on glycated Hb levels, average glycemia over 24 h, and other factors [9]. In addition, Vitamin D can directly promote the development of insulin receptors, increasing glucose uptake in human cells [10]. According to the gender-stratified analysis, a substantial association between gender and Vitamin D was established. Similarly, in a population-based cross-sectional study, it was discovered that middle-aged Caucasians had low levels of vitamin 25(OH) D3, which were independently linked to type 2 DM in females while not in males [10]. In women, the chance of having recently discovered or detected diabetes has more than doubled. The majority of the current type 2 diabetes recommendations for intensifying therapy following metformin are based on the possibility of additional advantages (such as weight loss) or a greater probability of side effects (e.g., hypoglycemia). While glucagon-like peptide-1 receptor agonists (GLP-1Ras) offer a similar reduction in severe adverse cardiac events, regardless of sex, sodium-glucose co-transport-2 inhibitors (SGLT-2Is) seem much less effective in treating diabetes in females than in males [11,12].

Additionally, adjustable social factors that are connected to a greater risk of type 2 diabetes and obesity, especially in females, include a lack of education, profession, and income. These factors also significantly contribute to unhealthy lifestyle activities and social inequities [13].

There is still a dearth of data on the prevalence of the clinical manifestation of DM. Furthermore, to diagnose DM in a timely manner, it is critical for the general population to comprehend the early clinical signs of the disorder. Therefore, this study evaluated the overall clinical manifestations of patients with type 2 DM with respect to gender.

## Materials and methods

This was a retrospective multicenter, cross-sectional study that was conducted at various hospitals, using a non-probability sampling technique. The duration of the study was about six months, from January 1, 2022, to June 30, 2022. The study included 590 type 2 diabetes patients, between the ages of 35 and 70. The study excluded participants with extreme weight loss, type 1 diabetes, low fasting glucose, low glucose tolerance, any surgery, and chemotherapy patients.

To identify patients with type 2 DM, the most recent HbA1c, a measure of glycemic control, was used. Age, gender, socioeconomic status, health status, co-morbidities, and diabetes symptoms were documented. Height and weight were also recorded for calculating the body mass index (BMI). Additionally, signs of depression, tension, and anxiety were evaluated. Blood pressure, breathing rate, and heart rate were all measured by researchers. The maximal blood pressure after three measurements was calculated along with the average of the pulse rate for the three readings. Data regarding current medical history and prior sleep disturbances (insomnia, abnormal actions or behaviors during sleep, and an inability to sleep at the desired time) were gathered using a questionnaire. Dry eyes were suspected based on a history of ocular pain, which included soreness, a gritty sensation, irritation, and inflammation, as well as the blurry vision that improved with blinking and copious amounts of tears. Random blood sugar readings were also taken, along with measurements of related biochemical markers such as triglycerides, total cholesterol, low-density lipoprotein cholesterol (LDL-C), and high-density lipoprotein cholesterol (HDL-C).

Data analysis was performed using SPSS Statistics v. 26.0 (IBM Corp., Armonk, NY, USA). Mean and standard deviations were determined for continuous variables. Many demographic parameters (like gender, and clinical features associated with type 2 diabetes) were recorded as frequencies and percentages. A chi-square test was applied to assess the association between overall symptoms associated with type 2 diabetes and gender. An independent t-test was applied to determine the significance level between means of demographic factors. A p-value of <0.05 is considered statistically significant. 

## Results

Patients with type 2 DM (n = 590) were included in the study. Of them, 280 (47.5%) were females and 310 (52.5%) were males. The male and female mean ages were 57.46±14.93 and 50.38±14.85 years, respectively, with a significant difference with gender (p<0.001). The male and female mean weights were 71.33±13.72 and 62.52±14.38 kg, respectively, with a statistically significant relationship between genders (p<0.001). The male and female mean heights were 68.72±10.78 and 65.23±9.95 inches, respectively, with a significant association between genders (p<0.001). Male and female BMIs were 25.54±11.94 and 24.20±8.45 kg/m^2^, with an insignificant association with genders (p=0.121). Male and female respiratory rates were 19.88±6.01 and 17.24± 4.93 breaths/min, respectively, with a significant association between genders (p<0.001). Male and female mean temperatures were 74.38±24.95 and 62.59±25.32 F, respectively, with a significant association between genders (p<0.001). The blood pressure of male and females were 177.74±47.66 and 163.51±45.73 mm Hg, respectively, with a significant association between genders (p<0.001), and the mean duration of male and female was 5.33±4.83 and 3.97±3.69 years respectively, with a statistically significant association between genders (p<0.001). The mean heart rates of males and females were 87.67±10.80 and 80.76±10.98 beats/min, respectively, with a significant association between genders (p<0.001). The mean number of cigarettes per day for males and females was 3.58±5.31 and 0.21±1.34, respectively, with statistically significant associations between genders (p<0.001). The duration of diabetes in males and females was 2.07±0.72 and 1.88±0.66 years respectively, with a significant association between genders (p=0.001). Male RBS was 285.82±106.91 and female RBS was 272.39±108.78, with an insignificant association observed between genders (p=0.131), as shown in Table 1.

**Table 1:** Demographic characteristics of type 2 patients with diabetes (n=590)

Variables	Male, Mean±SD	Female, Mean±SD	P-value
Age (years)	57.46±14.93	50.38±14.85	<0.001
Weight (kg)	71.33±13.72	62.52±14.38	<0.001
Height (inch)	68.72±10.78	65.23±9.95	<0.001
BMI (kg/m^2^)	25.54±11.94	24.20±8.45	0.121
Respiratory Rate (breath/min)	19.88±6.01	17.24±4.93	<0.001
Temperature ^o^F	74.38±24.95	62.59±25.32	<0.001
Systolic blood pressure mmHg	177.74±47.66	163.51±45.73	<0.001
Duration of hypertension (if yes), years	5.33±4.83	3.97±3.69	<0.001
Heart rate (beats/min)	87.67±10.80	80.76±10.98	<0.001
Smoking (if yes), number of cigarettes per day	3.58±5.31	0.21±1.34	<0.001
Duration of diabetes (years)	2.07±0.72	1.88±0.66	0.001
Random blood sugar (RBS)	285.82±106.91	272.39±108.78	0.131

The majority of male patients with diabetes 168 (54.2%) and female patients with diabetes 168 (60.0%) belonged to the middlmiddle classh a statistically significant association among them (p<0.001). Most males with diabetes 214 (69.0%) and female 174 (62.1%) patients had a history of hypertension; however, an insignificant association was observed between genders (p=0.078). There was a significant difference observed between genders with respect to dyslipidemia (p=0.004), depression (p<0.001), smoking (p<0.001), and physical activity (p<0.001), as shown in Table 2.

**Table 2:** Prevalence of comorbidities, age groups, and socioeconomic status with respect to gender

Variables		Male, n (%)	Female, n (%)	P-value
Socioeconomic status	Low	43(13.9%)	70(25.0%)	<0.001
	Middle	168(54.2%)	168(60.0%)	
	High	99(31.9%)	42(15.0%)	
History of hypertension	Yes	214(69.0%)	174(62.1%)	0.078
	No	96(31.0%)	106(37.9%)	
History of dyslipidemia	Yes	236(76.1%)	183(65.4%)	0.004
	No	74(23.9%)	97(34.6%)	
History of depression	Yes	122(39.4%)	71(25.4%)	<0.001
	No	188(60.6%)	209(74.6%)	
History of smoking	Yes	166(53.5%)	10(3.6%)	<0.001
	No	144(46.5%)	270(96.4%)	
Physical activity	Yes	140(45.2%)	177(63.2%)	<0.001
	No	170(54.8%)	103(36.8%)	

The occurrence of renal manifestation in type 2 patients with DM revealed that 112 (36.1%) male patients and 53 (18.9%) female diabetics urinate frequently, with a significant difference found between both genders (p<0.001). The majority of male diabetics 203 (65.5%) and female diabetics 217 (77.5%) urinate three times at night, with a significant difference between genders (p=0.005). Moreover, there was a significant difference found between genders in terms of the color of urine (p<0.001) and control of blood pressure (p=0.046), as shown in Table 3.

**Table 3:** The Distribution of renal manifestations in type 2 patients with diabetes with respect to gender

Variables		Male, n (%)	Female, n (%)	P-value
Frequent urination	Yes	112(36.1%)	53(18.9%)	<0.001
	No	198(63.9%)	227(81.1%)	
Urination at night	3 times at night	203(65.5%)	217(77.5%)	0.005
	at every two hour	97(31.3%)	58(20.7%)	
	at every hour	10(3.2%)	5(1.8%)	
Color of urine	Light-colored urine	199(64.2%)	228(81.4%)	<0.001
	Dark yellow urine	96(31.0%)	52(18.6%)	
	Very dark or bloody urine	15(4.8%)	0(0.0%)	
BP control becomes the worst	Yes	145(46.8%)	154(55.0%)	0.046
	No	165(53.2%)	126(45.0%)	

The prevalence of ocular manifestations in patients with DM revealed that flashes were observed in 53 (18.9%) female and 67 (21.6%) male patients, with a statistically insignificant difference found between both genders (p=0.419). Additionally, the distortion was observed in 67 (23.9%) female and 36 (11.6%) male patients, with a significant difference found between both genders (p<0.001). Moreover, a significant association was observed between genders relating to trouble reading or seeing faraway objects (p<0.001), and blurred vision (p=0.022). Furthermore, a statistically insignificant difference was observed between genders with respect to blind spots (p=0.350), night blindness (p=0.130), eye floaters (p=0.175), visual disturbances (p=0.175), and vision loss (p=0.259). Respiratory manifestations in patients with diabetes revealed that dyspnea was observed in 187 (60.3%) male patients with diabetes and 130 (46.4%) female patients with diabetes, with a significant association observed between genders (p=0.001). About 145 (46.8%) male and 115 (41.1%) female patients with diabetes-experienced dyspnea while climbing stairs, with a significant association observed between genders (p<0.001). Furthermore, there was a significant association observed between genders with respect to dyspnea severity (p<0.001), and severity of chest pain (p=0.019). Additionally, there was an insignificant association observed between genders with respect to chest tightness (p=0.796), as shown in Table 4.

**Table 4:** Distribution of ocular and respiratory manifestations in type 2 patients with diabetes with respect to gender

Variables		Male, n (%)	Female, n (%)	P-value
Flashes	Yes	67(21.6%)	53(18.9%)	0.419
	No	243(78.4%)	227(81.1%)	
Blind spots	Yes	103(33.2%)	83(29.6%)	0.350
	No	207(66.8%)	197(70.4%)	
Distortion	Yes	36(11.6%)	67(23.9%)	<0.001
	No	274(88.4%)	213(76.1%)	
Poor night vision/night blindness	Yes	81(26.1%)	89(31.8%)	0.130
	No	229(73.9%)	191(68.2%)	
Small dark spots eye floaters or streaks in vision	Yes	87(28.1%)	93(33.2%)	0.175
	No	223(71.9%)	187(66.8%)	
Trouble in reading or seeing faraway objects	Yes	162(52.3%)	104(37.1%)	<0.001
	No	148(47.7%)	176(62.9%)	
Visual disturbances	Yes	149(48.1%)	119(42.5%)	0.175
	No	161(51.9%)	161(57.5%)	
Blurry vision	Yes	96(31.0%)	112(40.0%)	0.022
	No	214(69.0%)	168(60.0%)	
Vision loss	Yes	136(43.9%)	110(39.3%)	0.259
	No	174(56.1%)	170(60.7%)	
Shortness of breath	Yes	187(60.3%)	130(46.4%)	0.001
	No	123(39.7%)	150(53.6%)	
Dyspnea grading	While climbing stairs	145(46.8%)	115(41.1%)	<0.001
	While walking for more than 6 h in a day	95(30.6%)	122(43.6%)	
	While walking for less than 6 hours in a day"	35(11.3%)	33(11.8%)	
	While at rest	35(11.3%)	10(3.6%)	
Dyspnea severity, if yes	Mild	79(25.5%)	141(50.4%)	<0.001
	Moderate	174(56.1%)	103(36.8%)	
	Severe	57(18.4%)	36(12.9%)	
Chest tightness	Yes	176(56.8%)	156(55.7%)	0.796
	No	134(43.2%)	124(44.3%)	
Severity of chest pain	Improves with rest	168(54.2%)	165(58.9%)	0.019
	Need pain relieving medication"	115(37.1%)	77(27.5%)	
	Requires hospital visit	27(8.7%)	38(13.6%)	

The occurrence of oral manifestations in type 2 patients with DM revealed a significant association between gender with respect to dry mouth (p<0.001), whereas an insignificant association was observed with respect to red, swollen, and painful gums (p=0.442), burning sensation (p=0.701), and sweet-smelling breath (p=0.631). Similarly, dermatological manifestations in patients with diabetes revealed that the majority of females with diabetes 216 (77.1%) had dry, cracked skin; compared with male diabetics 183 (59.0%), with a significant association observed between them (p<0.001). Moreover, a significant association was observed between genders with respect to light brown scaly patches (p=0.003), yellowish-reddish or brown patches on the skin (p=0.039), hard, thickened skin (p=0.010), and blisters (p<0.001). Furthermore, velvet-like dark skin was observed in 158 (56.4%) female diabetics and 155 (50.0%) male diabetics, with an insignificant association (p=0.118), as shown in Table 5.

**Table 5:** Distribution of oral and dermatological manifestations in type 2 patients with diabetes with respect to gender

Variables		Male, n (%)	Female, n (%)	P-value
Red, swollen, and painful gums	Yes	109(35.2%)	107(38.2%)	0.442
	No	201(64.8%)	173(61.8%)	
Dry mouth	Yes	50(16.1%)	88(31.4%)	<0.001
	No	260(83.9%)	192(68.6%)	
Burning sensation in the mouth	Yes	82(26.5%)	78(27.9%)	0.701
	No	228(73.5%)	202(72.1%)	
Sweet Smell breath	Yes	129(41.6%)	122(43.6%)	0.631
	No	181(58.4%)	158(56.4%)	
Dry cracked skin	Yes	183(59.0%)	216(77.1%)	<0.001
	No	127(41.0%)	64(22.9%)	
Light brown scaly patches	Yes	81(26.1%)	105(37.5%)	0.003
	No	229(73.9%)	175(62.5%)	
Yellow reddish or brown patches on the skin	Yes	104(33.5%)	117(41.8%)	0.039
	No	206(66.5%)	163(58.2%)	
A darker area of the skin that feels like velvet	Yes	155(50.0%)	158(56.4%)	0.118
	No	155(50.0%)	122(43.6%)	
Hard thickened skin	Yes	79(25.5%)	47(16.8%)	0.010
	No	231(74.5%)	233(83.2%)	
Blisters	Yes	46(14.8%)	79(28.2%)	<0.001
	No	264(85.2%)	201(71.8%)	

The prevalence of gastrointestinal and psychological manifestations in type 2 patients with DM revealed a significant association between genders with respect to increased thirst (p<0.001), increased hunger (p=0.003), delayed healing of wounds (p=0.017), cold sweating (p<0.001), mood swings (p=0.036), swelling of feet, ankles, hands or eyes (p=0.007), confusion or difficulty in concentration (p=0.022), burning pain in feet or legs (p<0.001), and muscular pain or cramps in legs or feet (p<0.001). Additionally, 226 (80.7%) female diabetics and 262 (84.5%) male diabetics reported fatigue, with an insignificant association between genders (p=0.223). Moreover, an insignificant association was observed between genders with respect to unexplained weight loss (p=0.672), appetite loss (p=0.420), time period of appetite loss (p=0.223), increased incidence and severity of infections (p=0.969), feeling tired (p=0.304), tingling or numbness in the hands or feet (p=0.300), too sensitive to touch feet (p=0.078), worsening symptoms at night (p=0.984), and insomnia (p=0.754), as shown in Table 6.

**Table 6:** The distribution of gastrointestinal and psychological manifestations in type 2 diabetic patients with respect to gender

Variable		Male, n (%)	Female, n (%)	P-value
Increased thirst	Yes	78(25.2%)	187(66.8%)	<0.001
	No	232(74.8%)	93(33.2%)	
Fatigue	Yes	262(84.5%)	226(80.7%)	0.223
	No	48(15.5%)	54(19.3%)	
Increased hunger	Yes	67(21.6%)	91(32.5%)	0.003
	No	243(78.4%)	189(67.5%)	
Unexplained weight loss	Yes	211(68.1%)	186(66.4%)	0.672
	No	99(31.9%)	94(33.6%)	
Loss of appetite	Yes	184(59.4%)	157(56.1%)	0.420
	No	126(40.6%)	123(43.9%)	
If yes, the mode of appetite loss	Sudden	145(46.8%)	117(41.8%)	0.223
	Insidious	165(53.2%)	163(58.2%)	
Increased incidence and severity of infections	Yes	139(44.8%)	126(45.0%)	0.969
	No	171(55.2%)	154(55.0%)	
Slow/delayed healing of wounds	Yes	212(68.4%)	216(77.1%)	0.017
	No	98(31.6%)	64(22.9%)	
Cold sweating	Yes	172(55.5%)	195(69.6%)	<0.001
	No	138(44.5%)	85(30.4%)	
Feeling tired and weak occasionally	Yes	222(71.6%)	211(75.4%)	0.304
	No	88(28.4%)	69(24.6%)	
Tingling or numbness in the hands or feet	Yes	201(64.8%)	170(60.7%)	0.300
	No	109(35.2%)	110(39.3%)	
Irritability or mood swings	Yes	213(68.7%)	214(76.4%)	0.036
	No	97(31.3%)	66(23.6%)	
Swelling of feet ankles hands or eyes	Yes	183(59.0%)	195(69.6%)	0.007
	No	127(41.0%)	85(30.4%)	
Confusion or difficulty in concentration	Yes	109(35.2%)	74(26.4%)	0.022
	No	201(64.8%)	206(73.6%)	
Burning pain in the legs or feet	Yes	155(50.0%)	181(64.6%)	<0.001
	No	155(50.0%)	99(35.4%)	
Too sensitive feet on touch	Yes	68(21.9%)	79(28.2%)	0.078
	No	242(78.1%)	201(71.8%)	
Muscular pain or cramps in the legs or feet	Yes	262(84.5%)	263(93.9%)	<0.001
	No	48(15.5%)	17(6.1%)	
Symptoms worsening at night	Yes	147(47.4%)	133(47.5%)	0.984
	No	163(52.6%)	147(52.5%)	
Insomnia	Yes	130(41.9%)	121(43.2%)	0.754
	No	180(58.1%)	159(56.8%)	

## Discussion

The occurrence of type 2 DM is rising, and its consequences, such as CHD, are increasing rapidly [14,15]. Therefore, this study demonstrated the clinical manifestation reported in type 2 patients with DM with respect to gender.

A study observed that the mean age of presentation of patients with diabetes was 50±11 years. Most of the patients with diabetes (37%) suffered diabetes for 10 years or longer, with the average patient having diabetes for 8.5 years [16]. This appears comparable to the Ahmed et al. study's finding that the mean age of patients with diabetes was 54 [17]. Also, reporting a comparable mean age of presentation Basit et al. The majority of them had long-term diabetes that affected their social and professional lives, as indicated by the mean age of DM presentation. [18] This study was consistent with the above-mentioned studies and revealed that the mean age of the male patient with diabetes was 57.46±14.93 years and that of the female patient with diabetes was 50.38±14.85 years. However, the results of the study revealed discrepancies in the duration of diabetes, showing that the mean duration of diabetes in men and women was 2.07±0.72 and 1.88±0.66 years, respectively.

Similarly, another research revealed that most of the participants in their investigation were women. Women were more likely to present in the OPD with dermatological symptoms, which may be a sign of increasing disease incidence and health concerns among women. [17] This study reported a slight preponderance of males 310 (52.5%) over female patients with diabetes 280 (47.5%). In terms of the dermatological manifestations, females were more likely than males to experience dry cracked skin, scaly patches, darker skin that looks like velvet, and blisters, with a significant relationship between them (p<0.05). 

In patients with DM, the overall incidence of skin infections ranged from 20% to 50% [19]. Patients with type 2 DM are particularly more likely to experience dermatological infections. Skin infections and gender were not related; however, individuals with poor glycemic control were shown to be more vulnerable to bacterial infections. Likewise, research by Basit et al. reported a greater frequency of infections in men. Additionally, greater exposure to pathogenic organisms and moist weather conditions may be responsible for this [18]. This study was not in accordance with the above-reported research and revealed that dermatological manifestations were noticed at a frequency ranging from 15% to 80%, and women were more affected by the skin manifestations. On the other hand, gender showed a statistically significant association (p<0.05).

Likewise, one study indicated that 5% of the patients with diabetes had thick skin indicative of diabetes [16]. This is brought on by collagen's non-enzymatic glycosylation, which reduces its solubility. The subcutaneous components' glycosylation progresses as the duration of diabetes lengthens. Other investigations have found a similar prevalence of thick skin [20]. The current study, which differed from the earlier research in that it found hard, thicker skin in patients with diabetes of both genders, 47 (16.8%) females and 79 (25.5%) males, had a statistically significant association (p=0.010).

Discrimination based on gender categories worsens environmental mental distress and stress reactions, particularly in women. According to certain studies, women are more likely to experience the negative cardio-metabolic effects of psychological stress, occupational stress, and sleep disturbances. This vulnerability may be partially attributed to poor behavior [21,22]. According to a sex-specific meta-analysis of epidemiological studies, Insomnia is 40% more likely to affect women of all ages [23]. In addition, obesity, and much more significantly insulin resistance-related (impaired glucose metabolism) conditions were linked to sleep disruption, reduced sleep, and poor sleep quality [24]. In another meta-analysis, insufficient sleep (<5 hr) and trouble staying asleep were both linked to an increased risk of diabetes. After gender categorization, however, equivalent impact estimates were seen in both genders [24]. This study supported the findings of the earlier research and found that somewhat more female patients with diabetes 121 (43.2%) than male patients with diabetes 130 (41.9%) complained of insomnia, indicating that sleep problems were more common in women.

Between men and women, smoking has a significant role. It has risen significantly among young women over the past decade, which could eventually lead to a rise in the prevalence of smoking-related diabetes in women [25]. According to a meta-analysis, the comparative threat of myocardial infarction, a serious and common complication in people with diabetes, caused by smoking seems 25% greater in females compared with males [26]. Likewise, according to a meta-analysis of cohort research, active and passive smoking are both associated with an increased risk of developing type 2 DM in both males and females, with an insignificant gender difference [26]. As far as this study is concerned, smoking is a significant risk factor that increases the chance of type 2 DM. There was a substantial difference between the genders among the male diabetics, with 144 (46.5%) of them smoking and 10 (3.6%) of them being female (p<0.001).

Another study conducted in Nigeria discovered sex-specific disparities in cardio-metabolic risk, microvascular, and macrovascular consequences in type 2 patients with diabetes. There were 210 (52.5%) female and 190 (47.5%) male individuals. The study population's average age was 60.6 + 9.93 years. Obesity and hypertension were more common in women. Contrary to earlier data [27], males were more likely than women to accomplish LDL treatment targets in type 2 DM in Nigeria (69.5% vs 59.0%, p<0.05). However, the mean total cholesterol was considerably greater in women; this conflicts with former research, on type 2 DM in Nigeria [28]. This study showed dissimilarity to the above-reported studies and revealed that there were 310 (52.5%) male and 280 (47.5%) female patients with diabetes with a mean age of 57.46±14.93 and 50.38±14.85 years respectively. Men had a higher incidence of hypertension 214 (69.0%) and dyslipidemia 236 (76.1%) than women. Moreover, in comparison to males, women had significantly higher levels of dyslipidemia (p=0.004).

Diabetes is a frequent cause of nocturia for a number of reasons. The flow of urine during the nighttime can be greatly increased by osmotic diuresis brought on by hyperglycemia [29]. One meta-analysis investigated the connection between nocturia and diabetes. First, among the 197,809 participants studied, diabetes approximately doubled the incidence of nocturia. Additionally, diabetes raised the incidence of nocturia in both males (p<0.00001) and females (p<0.0001) in the subgroup classification based on gender. Male subjects showed a greater correlation between diabetes and nocturia than female subjects [30]. In this study, nocturia was significantly associated with gender (p<0.05), with females 217 (77.5%) urinating more frequently than males 203 (65.5%).

## Conclusions

This study concluded that women with type 2 DM have a significantly higher incidence of muscular pain, urinary symptoms, neurological symptoms, and dermatological manifestations than men. In contrast, respiratory symptoms were significantly more pronounced in males than in females. The presence of comorbidities such as dyslipidemia significantly increased the probability of developing type 2 diabetes in both genders.
